# Mechanical-physicochemical properties and biocompatibility of catechin-incorporated adhesive resins

**DOI:** 10.1590/1678-7757-2018-0111

**Published:** 2019-01-07

**Authors:** Beatriz Maria Fonseca, Daphne Camara Barcellos, Tânia Mara da Silva, Alexandre Luis Souto Borges, Bruno das Neves Cavalcanti, Anuradha Prakki, Hueder Paulo Moisés de Oliveira, Sérgio Eduardo de Paiva Gonçalves

**Affiliations:** 1 Universidade Estadual Paulista (UNESP), Instituto de Ciência e Tecnologia, Departamento de Odontologia Restauradora, Grupo Acadêmico de Pesquisa Clínica, São José dos Campos, São Paulo, Brasil.; 2 University of Iowa, College of Dentistry and Dental Clinics, Department of Endodontics, Iowa City, Iowa, USA.; 3 University of Toronto, Faculty of Dentistry, Restorative Department, Toronto, Ontario, Canada.; 4 Centro de Ciências Naturais e Humanas, Universidade Federal do ABC, Santo André, São Paulo, Brasil.

**Keywords:** Dental adhesives, Dentin-bonding agents, Catechin, Collagen

## Abstract

Several anti-proteolytic dentin therapies are being exhaustively studied in an attempt to reduce dentin bond degradation and improve clinical performance and longevity of adhesive restorations.

**Objectives:**

This study assessed the effect of epigallocatechin-3-gallate (EGCG) on long-term bond strength when incorporated into adhesives.

**Material and Methods:**

Adhesive systems were formulated with EGCG concentrations of 0 wt%: (no EGCG; control); 0.5 wt% EGCG; 1.0 wt% EGCG, and 1.5 wt% EGCG. Flexural strength (FS), modulus of elasticity (ME), modulus of resilience (MR), compressive strength (CS), degree of conversion (DC), polymerization shrinkage (PS), percentage of water sorption (%WS), percentage of water solubility (%WL) and cytotoxicity properties were tested. Dentin microtensile bond strength (µTBS) was evaluated after 24 h and again after 6 months of water storage. The adhesive interface was analyzed using scanning electron microscopy (SEM).

**Results:**

No significant differences were found among the groups in terms of FS, ME, MR, CS and PS. EGCG-doped adhesives increased the DC relative to the control group. EGCG concentrations of 1.0 wt% and 0.5 wt% decreased the WS of adhesives. WL decreased in all cases in which EGCG was added to adhesives, regardless of the concentration. EGCG concentrations of 1.0 wt% and 0.5 wt% reduced cytotoxicity. EGCG concentrations of 1.0 wt% and 0.5 wt% preserved µTBS after 6 months of storage, while 1.5 wt% EGCG significantly decreased µTBS. SEM: the integrity of the hybrid layer was maintained in the 0.5 wt% and 1.0 wt% EGCG groups.

**Conclusion:**

EGCG concentrations of 1.0 wt% and 0.5 wt% showed better biological and mechanical performance, preserved bond strength and adhesive interface, and reduced cytotoxicity.

## Introduction

Immediate adhesive interface in dentin bond seems to be effective in dental restorations^1^, long-term bond strength values have been found to decrease significantly^2^. This decrease is due to the imperfect and degradable hybrid layer created by the current adhesive systems^3^. This degradation can be caused by factors, such as: the hydrophilic characteristics of infiltrated resin monomers^4^, and/or incomplete polymerization of infiltrated monomers, which can affect the chemical and mechanical stability of the hybrid layer^5^.

To reduce dentin bond deterioration and improve both clinical performance and longevity of adhesive restorations, several anti-proteolytic therapies are being exhaustively studied^6^ ^-^ ^12^. The incorporation of monomeric catechins found in polyphenols of green tea extracts, the most relevant of which is epigallocatechin-3-gallate (EGCG), is being tested in adhesives and in different adhesion protocols^10^ ^,^ ^11^ ^,^ ^13^.

EGCG has antioxidant and anti-inflammatory properties and is effective in inhibiting acid production in dental plaque bacteria; it also exhibits antimicrobial activity against *Streptococcus mutans* ^11^. EGCG engages in hydrophobic interactions with collagenases and gelatinases and can modify the secondary structure of MMPs by inhibiting their activity^11^ ^,^ ^14^. In addition to its anti-proteolytic activity, EGCG is also known to promote collagen cross-linking through hydrogen bonding, thus improving collagen properties such as modulus of elasticity^9^. A hydrogen bonding interaction between EGCG and Bis-GMA hydroxyl groups may also occur. At concentrations higher than 2% w/w, EGCG has been shown to impair the degree of conversion of monomers^10^. EGCG has been shown to be a promising agent in the maintenance of long-term dentin bond strength^10^ ^,^ ^13^. The incorporation of EGCG into adhesive systems is one of several clinical strategies that seek to preserve the longevity of composite restorations. However, changes in the composition of adhesive systems may involve deleterious mechanical, physical, and chemical changes in their material properties^10^.

Thus, this study evaluated the long-term bond interface of EGCG-doped etch-and-rinse adhesive systems as determined by *in vitro* cytotoxicity through tests on human dental pulp fibroblasts and by adhesive properties (bond strength, flexural strength, modulus of elasticity, modulus of resilience, compressive strength, degree of conversion, polymerization shrinkage, water sorption and water solubility). Bond strength was evaluated after 24 hours and again after 6 months of water storage. The null hypotheses tested were: experimental adhesives can achieve similar bond strength when compared to control adhesives (no EGCG), storage time does not affect the bond strength of model adhesives, and experimental adhesives can achieve similar adhesive properties and cytotoxicity when compared to control adhesives.

## Material and methods

### Experimental adhesive system preparation

The model adhesives consisted of 45 wt% bisphenol-A diglycidyl ether dimethacrylate (Bis-GMA) and 55 wt% 2-hydroxyethyl methacrylate (HEMA), as it is common among the monomers used in dentin adhesives^15^. The photoinitiators used were 0.5 wt% of camphorquinone (CQ), which served as hydrophobic photosensitizer, and 0.5 wt% of 2-(dimethylamino) ethyl methacrylate (DMAEMA), which served as hydrophilic co-initiator (Sigma-Aldrich, St Louis, Missouri, USA)^15^. The neat adhesive system was prepared in brown glass vials and shaken for 48 h to form a homogeneous solution^9^ ^,^ ^15^.

EGCG (Sigma-Aldrich, St Louis, Missouri, USA) was added into the neat adhesive system at different concentrations. The formulation groups were as follows:

Control Group: control dentin adhesive (without EGCG);

0.5 wt% Group: EGCG-doped adhesive system with 0.5 wt% incorporation of EGCG;

1.0 wt% Group: EGCG-doped adhesive system with 1.0 wt% incorporation of EGCG;

1.5 wt% Group: EGCG-doped adhesive system with 1.5 wt% incorporation of EGCG.

Shaking in the dark for 10 min at 2000 rpm was required to yield well-mixed adhesive resin solutions^7^.

### Cytotoxicity

In the cytotoxicity test, the fibroblasts of a germ from a human third molar (FP7 cell line) were cultured in DMEM supplemented with 10% fetal bovine serum (FBS; Thermo Fischer Scientific, Waltham, Massachusetts, USA) and 1% antimycotic-antibiotic solution (10,000 units of penicillin, 10 mg of streptomycin, and 25 µg of amphotericin B *per* mL in 0.9% sodium chloride (Sigma-Aldrich, St. Louis, Missouri, USA) at 37°C and 5% CO_2_. Cultures were supplied with fresh medium every 2 days^7^ ^,^ ^16^. A total of 3×10^3^ cells were placed in the experimental adhesive system in each well of the 96-well plates before incubation for 24 h at 37°C (5% CO_2_).

Tubes containing 0.4 g of the different adhesive groups were filled with 1 ml of fresh DMEM in order to produce the conditioned medium. The medium was applied to the uncured adhesives and agitated for 1 min to achieve homogenization^16^. After 24 h, the media were removed, and the cell cultures were exposed to 100 µl of serial dilutions (10%, 1%, 0.1%, 0.01%), 100 µl of culture medium with cells (positive control), and 100 µl culture medium without cells (blank – negative control). The plates were incubated for 24 h, 48 h, and 72 h in a 37°C incubator (5% of CO_2_).

Cellular proteins were marked by adding a solution consisting of protein dye sulforhodamine B (SRB) and 0.4% acetic acid (1%), followed by incubation for 30 min at room temperature. The SRB solution was removed, and the plates were washed 5 times with 1% acetic acid before air drying. Bound SRB was resolubilized with unbuffered Tris-buffer 10 mM solution^16^. The absorbance peak was read at a wavelength of 570 nm. The percentage of viable cells in each well was determined and normalized for negative control statistical analysis. Absorbance of the positive control (cells grown only in DMEM media) represents 100% survival.

The mean percentages of viable cells were analyzed using the Kruskal-Wallis test and Dunn's multiple comparison test (α=5%).

### Degree of conversion

The degree of conversion was monitored *in situ* using an infrared spectrometer (FTIR, Spectrum 400; Perkin-Elmer, Waltham, Massachusetts, USA) with a resolution of 4 cm^-1^ in the ATR sampling mode^15^. 10 μl of experimental adhesive model was placed on the ATR crystal, and a transparent coverslip, attached using a piece of tape, was placed on the sample to prevent evaporation of components. The adhesive samples were light cured for 20 s using a photocuring unit LED light curing system (Demi Plus; Kerr Manufacturing Company, Orange, California, USA), with a power density of 1100 mW/cm^2^. A time-resolved spectrum collector (Spectrum TimeBase, Perkin-Elmer, MA, USA) was used for continuous and automatic collection of spectra during polymerization.

To determine the degree of conversion, spectra of a droplet of uncured adhesives and polymerized adhesives were acquired over a spectral range of 4000 to 650 cm^-1^. The change in the band height ratios of the aliphatic carbon-carbon double bond (peak at 1638 cm^-1^) and the aromatic C=C (peak at 1608 cm^-1^) (phenyl) in both cured and uncured states were monitored^15^ ^,^ ^17^. The formula used to calculate the degree of conversion relied on the decrease in the intensity band ratios before and after light curing (Equation A.1). All experiments were carried out in triplicate, and the results were averaged. Mean values were analyzed using one-way ANOVA and the Tukey test (α=5%).

Equation A.1 - %Degree of conversion $DC\left(\%\right)=\left(1–\left(\frac{Rcured}{Runcured}\right)\right)\times 100$

### Flexural strength, modulus of elasticity, and modulus of resilience

Ten specimens from each group were prepared using the method presented by Barcellos, et al.^7^ (2016), which relied on rectangular silicon molds (12 mm length × 2 mm width × 2 mm height; ISO 4049:2009). Uncured adhesive was dropped onto the molds, covered with a Mylar strip, and light cured from the top surface for 40 s: 1100 mW/cm^2^; LED Light Curing System, (Demi Plus; Kerr Manufacturing Company, Orange, California, USA) at 2 different locations (20 s from the right; and 20 s from the left). The bottom surface was also light cured for an additional 20 s. Specimens were stored for 24 h in distilled water at 37°C prior to testing^18^.

Flexural properties were evaluated using a three-point flexural strength test performed with a universal testing machine (EMIC DL-200MF; Equipamentos e Sistemas Ltda, São José dos Pinhais, Paraná, Brazil) at a crosshead speed of 0.5 mm/min^18^. Flexural strength was determined as the load at the fracture point, and the modulus of elasticity was calculated based on recorded load deflection curves^10^. Coefficients of variation for the modulus of resilience were calculated using the data on flexural strength and modulus of elasticity in the formula described in Equation A.2, where FS is the flexural strength (in MPa), ME is the modulus of elasticity (in MPa), and RM is modulus of resilience (in MPa). Mean flexural strength (in MPa), modulus of elasticity (in MPa), and modulus of resilience (in MPa) values were analyzed using one-way ANOVA and the Tukey's test (α=5%).

Equation A.2 - Modulus of resilience $RM=\frac{{\left(FS\right)}^{2}}{2x\left(ME\right)}$

### Compressive strength (CS)

Ten specimens from each group were prepared using a silicon mold (3.0 mm diameter × 6.0 mm height)^19^. Three layers of uncured adhesive were dropped onto the silicon mold and light cured: 1100 mW/cm^2^; LED light curing system (Demi Plus; Kerr Manufacturing Company, Orange, California, USA) for 20 s for each layer. The last layer was covered with a Mylar strip and a glass slide, then it was light cured for 20 s. Additional light curing was performed for 20 s on the opposite side and on each lateral face of the cylinder after the silicone mold was removed. Specimens were stored in distilled water at 37°C for 24 h prior to testing^7^. They were then evaluated under compressive load in a universal testing machine with a crosshead speed of 1 mm/min. Mean compressive strength values (in MPa) were analyzed using one-way ANOVA and the Tukey test (α=5%).

### Percentage of water sorption and water solubility

Ten disc-shaped specimens from each adhesive group were fabricated using a silicon mold (6.0 mm diameter × 2.0 mm height). Uncured dentin model adhesive was placed in the silicon mold, a Mylar strip and a glass slide were placed onto it, and the adhesive was light cured for 20 s^7^. Additional light curing was performed for 20 s on the bottom of the specimen^7^.

Specimens were stored in a desiccator containing freshly dried silica gel. After 24 h, they were weighed using a 0.0001 mg precision scale (Mettler Toledo, Columbus, Ohio, USA). This cycle was repeated until a constant mass (m_i_) was obtained. The specimens were immersed in 1 ml of distilled water at 37°C for 28 days^20^. Every 24 h, the specimens were removed, blotted dried, re-weighed (m_s_), and returned to the water. After 28 days, the specimens were again dried inside the desiccator and weighed daily until a constant mass was achieved (m_d_). Water sorption and water solubility were calculated using the formula presented in Equations A.3 and A.4 ^21^. For each test, mean values were analyzed using one-way ANOVA and the Tukey's test (α=5%).

Equation A.3 - %Water sorption $WS\left(\%\right)=\left(\frac{m_{s}–m_{i}}{m_{i}}\right)\times 100$

Equation A.4 - %Water solubility $WL\left(\%\right)=\left(\frac{m_{i}–m_{d}}{m_{i}}\right)\times 100$

### Polymerization volume shrinkage

The polymerization volume shrinkage of the model adhesives was measured using an accurate volumetric shrinkage instrument Acuvol^™^ (Bisco Dental Products, Schaumburg, Illinois, USA). A total of 2 µl of each adhesive was placed on the equipment support. For 15 s of the 20 s curing processes, a camera captured images (10 reads) of the drop^7^. This allowed for a comparison of the drop volume before and after light curing. The mean volume change (percentage) after light curing was calculated for each group. Mean values were analyzed using one-way ANOVA and the Tukey test (α=5%).

### Microtensile bond strength (µTBS)

The study was approved by the Local Review Board (n° 11.794). Eighty sound human molars that had been extracted for therapeutic reasons were used in this study. Flat mid-coronal dentin surfaces were exposed by using water-cooled 450-grit aluminum oxide abrasive discs (Extec Corp., Enfield, CT, USA) in a polishing device (Panambra, São Paulo, SP, Brazil). Next, surface smear layers were standardized through polishing using 600-grit aluminum oxide abrasive discs (Extec Corp., Enfield, CT, USA) for 30 s under water cooling.

For the µTBS test, 30 wt% of 99% ethanol was added to the model dentin adhesives and shaken for 3 min at 2000 rpm. The restorative procedure was the same for all of the experimental groups (n=20). Dentin surfaces were etched with 37% phosphoric acid gel for 15 s and rinsed. The excess moisture was removed with absorbent paper. Two layers of each evaluated dentin adhesive were actively applied on demineralized dentin surfaces for 20 s, gently air dried for 10 s, and light cured for 20 s (1100 mW/cm^2^ LED light curing system, DEMI Plus, Kerr Manufacturing Company, Orange, California, USA). Nanocomposite resin blocks (Filtek Z350, 3M ESPE, St. Paul, MN, USA) were built up on the bonded surfaces and light cured for 20 s at each increment according to the manufacturer's instructions. All restored samples were stored in distilled water at 37°C.

Half of the teeth from each group was stored in distilled water at 37°C for 6 months before testing. The other half was tested after 24 h of water storage to determine µTBS. The samples were sectioned into dentin-resin sticks (sections measuring approximately 1 mm), which produced 5 testing sticks *per* tooth. The sticks were attached to a microtensile device in a universal testing machine (EMIC (Equipamentos e Sistemas Ltda, São José dos Pinhais, Paraná, Brazil) at a crosshead speed of 0.5 mm/min and using a 10 kg load cell. They were fractured in accordance with ISO 11405:1994.

The failure modes were analyzed under a stereomicroscope (Karl Zeiss, Oberkochen, Baden-Württemberg, Germany) and classified as adhesive, mixed, cohesive in dentin, or cohesive in composite resin. Only adhesive and mixed failures were included in the statistical analysis. The mean values (in MPa) for the beams originating from each tooth were used for the statistical analysis. Data were analyzed using two-way ANOVA (adhesives model; storage time) and the Tukey test (α=5%).

### Scanning electron microscopy (SEM)

Two teeth from each group were used in the SEM analysis in order to analyze the adhesive interface. After the restorative procedure for the microtensile bond strength test, the teeth were sectioned perpendicular to the bonding interface (EMIC; Equipamentos e Sistemas Ltda, São José dos Pinhais, Paraná, Brazil). Samples were polished with aluminum oxide abrasive discs (600, 1200 and 4000) in a polishing device under water cooling. Next, samples were fixed with 2.5% glutaraldehyde in a 0.1 M sodium cacodylate buffer solution at 4°C for 12 h with 3 exchanges, followed by distilled water for 1 min. They were then dehydrated in an ascending series of ethanol solutions (25% for 20 min, 50% for 20 min, 75% for 20 min, 95% for 30 min and 100% for 60 min). Next, they were immersed in hexamethyldisilazane (Fluka) in a gas exhaust hood for 10 min. They were then placed on a filter paper under a glass bell for 30 min at room temperature to complete the dehydration process^8^ ^,^ ^22^. Finally, samples were mounted on aluminum stubs and examined using SEM ProX (Phenom World, Eindhoven, Noord-Brabant, Netherlands) under low vacuum and at 2000× magnification.

## Results

Mean bond strength values obtained for each group at different storage times are shown in Table 1. Bond strength was affected by adhesives (F=3.20; p=0.028) and storage time (F=34.91; p=0.000). Interactions were also significant (F=7.10; p=0.000). All experimental model adhesives exhibited similar bond strength values at 24 h. After 6 months of storage in water, bond strength did not decrease in the tests involving 0.5 wt% EGCG and 1.0 wt% EGCG adhesives, while the control group and the group with 1.5 wt% EGCG exhibited significantly lower bond strength values (p>0.05). With regard to fracture modes, the percentage of adhesive failures at the 6 months mark was pronounced in the 1.5 wt% EGCG adhesive (95% of adhesive failure) and in the control adhesive (90% of adhesive failure).

**Table 1:** Mean values (± standard deviation) of bond strength and the results of Tukey test for adhesives and storage times

Model adhesives	Storage Time	Mean (± SD)		Homogeneous groups*	
Control	24 h	27.15 (± 4.20)	A		
0.5 wt% EGCG	24 h	27.03 (± 2.72)	A		
1.5 wt% EGCG	24 h	24.93 (± 4.55)	A		
0.5 wt% EGCG	6 m	23.00 (± 3.84)	A	B	
1.0 wt% EGCG	24 h	22.41 (± 4.17)	A	B	
1.0 wt% EGCG	6 m	22.07 (± 4.34)	A	B	
Control	6 m	17.63 (± 2.51)		B	
1.5 wt% EGCG	6 m	15.00 (± 2.91)			C

* Same letters indicate no statistical differences among groups (p<0.05)

The mean flexural strength (FS), modulus of elasticity (ME), modulus of resilience (MR), compressive strength (CS), percentage of water sorption (%WS), percentage of water solubility (%WL), polymerization shrinkage (PS), and degree of conversion (DC) values for each group are shown in Table 2. The groups did not differ significantly in relation to FS, ME, MR, CS, or PS (p>0.05). Incorporation of 0.5 wt% EGCG significantly decreased the %WS when compared to the control adhesive (p=0.010). Incorporation of 0.5 wt% and 1.0 wt% EGCG significantly decreased WL when compared to the control sample (p=0.001). The DC for all of the adhesives containing EGCG was in the range of 77%. Incorporation of EGCG significantly increased the DC relative to that of the control group (p=0.0002).

**Table 2:** Mean values ± standard deviation of flexural strength (FS), modulus of elasticity, (ME), modulus of resilience (RM), compressive strength (CS), water sorption (%WS), water solubility (%WL), degree of conversion (%DC) and polymerization shrinkage (%PS) values of model adhesives

	Control	1.5wt% EGCG	1.0wt% EGCG	0.5wt% EGCG
FS (MPa)	89.37±5.18^a^	85.15±5.18^a^	95.80±5.18^a^	97.54±4.94^a^
ME (GPa)	0.82±.04^a^	0.85±0.4^a^	0.88±.0.04^a^	0.87±0.04^a^
RM (MPa)	4.89±0.87^a^	5.31±1.18^a^	5.23±1.01^a^	4.25±1.68^a^
CS (MPa)	275.21±19.12^a^	257.46±17.10^a^	247.14±17.10^a^	251.23±17.10^a^
%WS	0.009±0.00^a^	0.009±0.00^a^	0.009±0.00^a^	0.008±0.00^b^
%WL	0.23±0.03^a^	0.18±0.04^ab^	0.13±0.03^bc^	0.10±0.02^c^
%DC	68.42±2.79^b^	78.01±1.98^a^	77.91±1.56^a^	77.61±0.66^a^
%PS	21.07±2.07^a^	24.34±2.27^a^	18.82±2.07^a^	18.09±2.27^a^

Same letters within same column indicate no statistical difference among groups (p<0.05)

**Figure 1:**
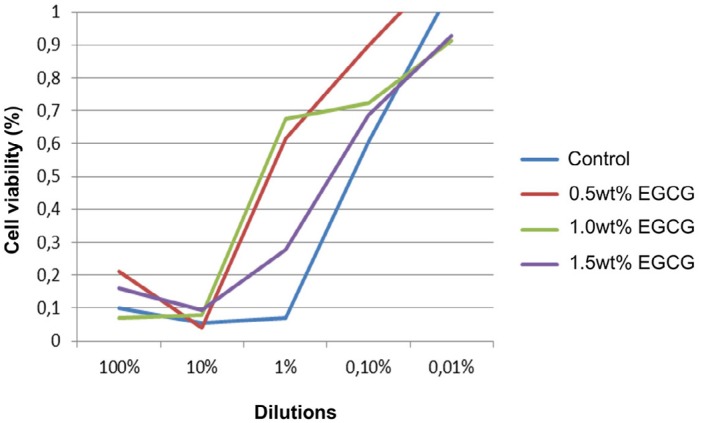
Graph of the viability curves (in percentages) of fibroblasts cells in serial dilutions of the adhesives tested

The viability curves (in percentages) of FP7 cells in serial dilutions of the adhesives tested are presented in Figure 1. There were statistically significant differences between the cytotoxicity results of the adhesives tested (p=0.005). The 0.5 wt% EGCG and 1.0 wt% EGCG adhesives presented significantly higher cell viability when compared to the control adhesive in the case of the medium with 1% dilution (Table 3).

**Table 3:** Means and standard deviation of %viable cells for the model adhesives tested

Model adhesives	Median (25°-75°)	Homogeneous groups*		
1.0 wt% EGCG	70.6 (51.1 – 80.5)	A		
0.5 wt% EGCG	57.7 (44.7 – 81.7)	A		
1.5 wt% EGCG	28.1 (25.3 – 29.7)	A		B
Control	7.4 (5.5 – 7.9)			B

* Same letters indicate no statistical difference among groups (p<0.05)

**Figure 2:**
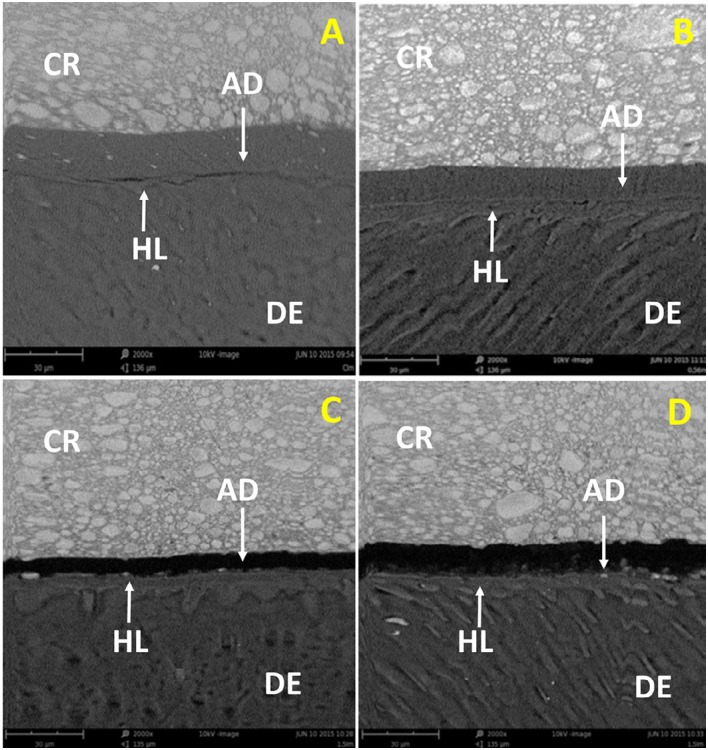
Baseline photomicrographs of the bonding interface between dentin (DE) and composite resin (CR): A) Control group; B) EGCG 0.5 wt%; C) EGCG 1.0 wt%; D) EGCG 1.5 wt%. AD=adhesive; HL=hybrid layer

**Figure 3:**
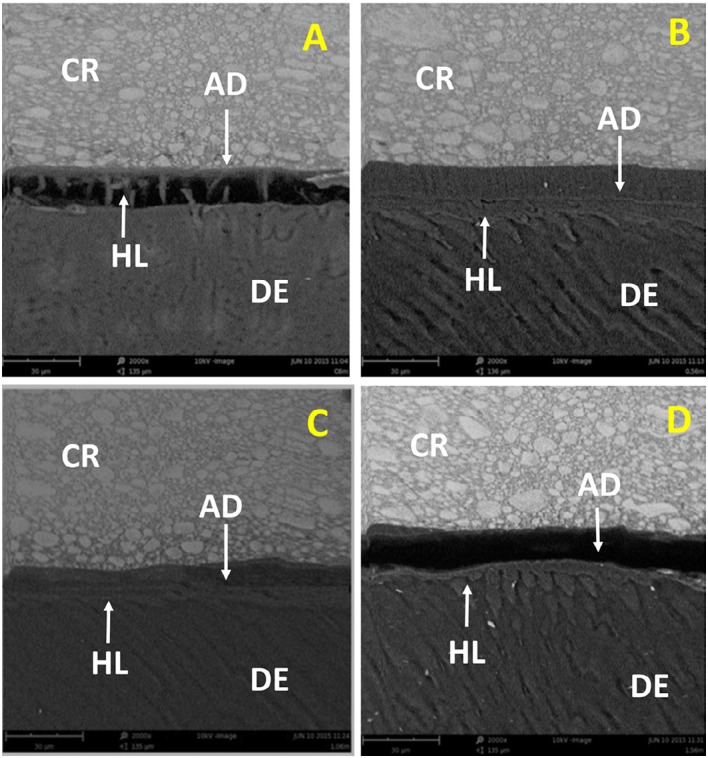
Photomicrographs of the bonding interface between dentin (DE) and composite resin (CR) after 6 months of storage: A) Control group; B) EGCG 0.5 wt%; C) EGCG 1.0 wt%; D) EGCG 1.5 wt%. AD=adhesive; HL=hybrid layer

The SEM analysis showed that all experimental adhesives were able to produce a hybrid layer with some resin tags inside dentinal tubules and a continuous thin layer of adhesive (Figure 2). After 6 months of water storage, a crack was observed in the control group, and a gap was observed between the adhesive layer and the hybrid layer in the 1.5 wt% EGCG group. The 0.5 wt% EGCG and 1.0 wt% EGCG groups maintained the integrity of the hybrid layer with no failures or cracks (Figure 3).

## Discussion

Incorporation of 0.5 wt% EGCG in dentin model adhesives resulted in higher bond strength values when compared to 1.5 wt% EGCG at the 6-months evaluation; the first null hypothesis is therefore rejected. The 1.5 wt% EGCG and control adhesives decreased bond strength after 6 months of water storage, a finding which disproves the second null hypothesis. Bond strength after 24 h did not differ significantly between the dentin model adhesives that were EGCG-doped with 0.5 wt%, 1.0 wt%, and 1.5 wt%. Although they provided adequate immediate adhesion, the current adhesive systems have been shown to result in progressive and long-term degradation of the hybrid layer^23^. This deterioration occurs through the hydrolysis process caused by the exogenous water from the oral environmental and/or endogenous water from the pulp fluid, which induces phase separation of the adhesives; it may also be caused by the activity of dentin proteolytic enzymes such as MMPs, which act directly upon the uncovered collagen fibrils on the bottom of the hybrid layer, especially when the bounded water does not evaporate during the adhesive protocol^5^ ^,^ ^6^ ^,^ ^18^ ^,^ ^23^ ^-^ ^26^.

It has been suggested that inhibition of MMPs by EGCG occurs when links with catalytic or allosteric sites of the enzymes alter their conformation^25^ or through a zinc chelating effect^7^ ^,^ ^27^. The molecular structure of EGCG also suggests a mechanism of interaction with proteins^28^. The phenolic component of EGCG contains a phenyl with hydroxyl groups (-OH) and has a phenol function. This property is the result of a combination of the hydrophobic nature of the aromatic group and the hydrophilic nature of the polar hydroxyl substituent. Hydrophobic moiety induces linkages between Van der Waals forces and other hydrophobic homologous molecules, while the hydrophilic portion links through hydrogen bonding. This bi-functional nature is responsible for the physical interaction between phenolic compounds and proteins^28^. EGCG has hydrophobic and hydrophilic interactions with MMPs, which causes a change in the secondary structure, thus inhibiting their action^13^.

Hirashi, et al.^20^ (2013) used EGCG as a cross-linking agent in a solution applied after acid conditioning; our results, therefore, corroborate their findings, EGCG can augment mechanical properties and resistance to proteolytic degradation, even when incorporated into an adhesive system. Monomeric catechins with a galloyl radical, such as EGCG, are more effective in increasing the collagen modulus of elasticity and in reducing enzymatic degradation by inhibiting MMP-9 activity. This indicates a correlation between the stability of collagen and specific chemical structures present in the monomeric compounds^29^.

However, the incorporation of 1.5 wt% EGCG into the experimental adhesive was not capable of stabilizing the hybrid layer, which exhibited an interface bond strength value similar to that of the control group after 6 months. It could be speculated that the EGCG concentration of 1.5 wt% may have interfered with the chemical interaction between the resin monomers and collagen fibrils, damaged the formation of the hybrid layer or led to a high degree of conversion (78%) but with the inappropriate formation of linear polymer chains^13^, thus resulting in an adhesive with low stability in wet environment.

The degree of conversion is the main chemical property of dental materials from a clinical perspective^7^. EGCG-doped adhesives exhibited higher degrees of conversion than those in the control group (Table 2). Furthermore, the values observed in the control group are consistent with those presented by Ye, et al.^15^ (2009), who used the same components in a manipulated adhesive (degree of conversion of approximately 70%). Du, et al.^13^ (2012) analyzed Single Bond-doped with EGCG at concentrations of 0.5 wt%, 1.0 wt%, and 1.5 wt%. The authors concluded that the degree of conversion of the adhesive systems was not significantly affected by the incorporation of EGCG at different concentrations. Similar to the findings reported by Du, et al.^13^ (2012) and Pallan, et al.^10^ (2012), the present study found that EGCG did not affect the degree of conversion of adhesive monomers.

The physical and mechanical properties of adhesives strongly depend upon the degree of conversion^30^. Therefore, higher values of mechanical and physical properties were expected for EGCG-doped adhesives due to the possible hydrogen bond established between EGCG and Bis-GMA. However, the results showed that the EGCG-doped adhesives did not differ significantly at the different concentrations. The samples reached a flexural strength, modulus of elasticity, modulus of resilience, compressive strength and percentage of polymerization volume shrinkage comparable to that of the control adhesive (Table 2). These results partially disprove the third null hypothesis.

Certain properties observed herein are likely to reflect the intrinsic bonds that EGCG can establish with monomeric components. The structure of Bis-GMA is rigid and viscous, and the different densities of cross-linking among the groups were unable to alter most of the mechanical and physical properties studied. A possible hydrogen bonding interaction between EGCG and Bis-GMA hydroxyl groups is expected to occur. Through not a scope of this study, the chemistry of the interaction EGCG-adhesive monomers interaction deserves to be investigated in further research.

The results presented in this study are consistent with Neri, et al.^30^ (2014), who observed that adhesives with EGCG concentrations at 0.01 wt% and 0.1 wt% did not differ in flexural strength or percentage of water sorption of the adhesives, which showed no differences in their physical or chemical properties at these different concentrations. EGCG is known to promote collagen cross-linking through hydrogen bonding, thus improving collagen properties such as modulus of elasticity^9^.

Restorative material that is highly resilient can change, deform or flex dissipating incoming voltages and is, therefore, better able to help preserve the adhesive bond between the tooth and the restoration^31^. This dissipation preserves the adhesive interface and can support distortions that occur due to microscopic movements of dental substrates^32^, causing them to behave as a single body. Adhesives in which EGCG was incorporated did not differ statistically from the control sample in terms of modulus of resilience. In other words, the incorporation of EGCG did not affect the ability of the material to bend or deform, nor did it affect its ability to dissipate tensions occurring in the interface.

The water sorption and solubility phenomena of adhesive systems can create undesirable changes in structure and can interfere with the function of adhesives. The association between Bis-GMA (resin organic layer) and HEMA (aqueous phase) polymers and water-soluble particles, creates droplets within the aqueous sample, and this diffusion extends along the osmotic gradient. The balance is achieved only when the osmotic stress and the elastic polymer stabilize^10^. The water sorption by the polymer may be associated with the hydrophilicity of resin monomers^33^. Incorporation of 0.5 wt% EGCG resulted in significantly lower percentage of water sorption and solubility in the model adhesive. This may have occurred due to the presence of EGCG, which may reduce hydrophilicity. Further in-depth studies are needed to investigate the chemical reaction that occurs between the Bis-GMA/HEMA monomers and EGCG molecule to understand a possible protective effect of EGCG on the water sorption of adhesives.

Simplified adhesives usually have a high percentage of water solubility^33^. They often have negative effects on the structure and function of the polymer matrix and may aid in degrading the dentin bond, causing premature failure of the restoration. Incorporation of 0.5 wt% and 1.0 wt% EGCG significantly decreased the percentage of water solubility. It is possible that this reduction, when combined with the anti-proteolytic activity of EGCG, may have contributed to maintaining the bond interface of these adhesives after 6 months of water storage, as was also observed in the SEM analysis. The same percentage of water solubility results was not observed in the 1.5 wt% EGCG and control groups. Subsequently, this result may have influenced on the reduction in the bond strength values after 6 months of water storage. The water present in the saliva, in the intrinsic wetness of dentin, in the bonding technique, and as a result of the hydrophilic nature of simplified adhesives, all play a role in solubilizing resin polymers, separating polymeric chains, and limiting the effects of the adhesive system's physical and mechanical properties at the bond interface^17^. However, the 0.5 wt% and 1.0 wt% EGCG concentrations were shown to be the best ones for promoting cross-linking between Bis-GMA chains and also with collagen fibrils, avoiding bound water into collagen fibrils due to its lower hydrophilicity^24^.

The cytotoxicity analysis in this study showed that the 0.5 wt% EGCG and 1.0 wt% EGCG concentrations are less cytotoxic than the control adhesive. Biologically, the hybrid layer can seal the tooth-restoration interface and protect pulpal tissue^34^. However, adhesives can release compounds, which can diffuse through the subjacent dentinal tubules and reach the pulpal tissue, a process which can have biological effects with toxic potential.

Figure 1 shows that, at 10% dilution of the adhesives into the culture media, cell growth for all adhesives was less than 10% (0.1 cell viability). However, at 1.0% dilution of adhesives into the culture media, 0.5 wt% EGCG and 1.0 wt% EGCG adhesives enabled more than 50% fibroblast cell growth, a result which is suggestive of low cytotoxicity for these adhesives. The high cell viability values for the 0.5 wt% EGCG and 1.0 wt% EGCG concentrations relative to the control group could be explained by the fact that, at certain levels, catechins have been found to have excellent biocompatible and chemopreventive properties; for example, they are able to protect normal cells against genotoxic effects^34^. On the other hand, the 1.5 wt% EGCG adhesive was incapable of providing the positive effects on fibroblasts observed in the 0.5 wt% EGCG and 1.0 wt% EGCG adhesives; its results were similar to those of the control sample.

Zarella, et al.^35^ (2003) found that 1.0 wt% EGCG was not cytotoxicity to odontoblast-like cells and retained its anti-proteolytic activity after extraction from a dental copolymer, results which are consistent with those of this study. In another study^26^, EGCG was found to modulate secretion of various inflammatory and anti-inflammatory mediators in odontoblastic cells. The authors analyzed smaller EGCG concentrations (2.5 to 160 µM) in cytotoxicity tests than those used in this study (0.5 wt%=10.9 mM; 1.0 wt%=21.8 mM; 1.5 wt%=32.7 mM). However, even at high concentrations, it should be argued that the EGCG does not show a relevantly cytotoxic behavior.

Considering the results presented and their consistency with the literature, it can be stated that EGCG incorporated into manipulated adhesive systems does not interfere in polymerization and, as a consequence, produces better results when at the concentrations of 0.5 wt% and 1.0 wt%. At these concentrations, no cytotoxic effects were observed, better results were obtained in the physical and mechanical analyses, and long-term bond strength was achieved through hydrolytic degradation of monomer resins after 6 months. Due to the limited scope of the study, further studies should be conducted in order to clarify the chemical interaction that occurs in the incorporation of EGCG particles in adhesive monomers, information which would complement the results presented herein.

## Conclusions

According to the results obtained, it can be concluded that: the incorporation of EGCG in experimental adhesive systems at concentrations of 0.5 wt% and 1.0 wt% produced adhesives with better biological and mechanical performance and that EGCG is therefore a potentially useful component in adhesives that offer long-term bond integrity.
